# Chronic Inhalation of Sulphureous Water Mitigates Early Amyloid Pathology and Associated Microglial Changes in 5XFAD Mice

**DOI:** 10.3390/brainsci16101069

**Published:** 2026-10-05

**Authors:** Martina Bodria, Eleonora Daini, Stefano Pioli, Monica Piemontese, Eleonora Vandini, Alessandra Ottani, Michele Zoli, Daniela Giuliani, Antonietta Vilella

**Affiliations:** 1 Department of Biomedical, Metabolic and Neural Sciences, Section of Physiology and Neurosciences, University of Modena and Reggio Emilia, 41125 Modena, Italy; martina.bodria@unimore.it (M.B.); eleonora.daini@unimore.it (E.D.); monica.piemontese@unimore.it (M.P.);; 2 Department of Biomedical, Metabolic and Neural Sciences, Section of Pharmacology and Molecular Medicine, University of Modena and Reggio Emilia, 41125 Modena, Italy; doc.stefano.pioli@gmail.com (S.P.); eleonora.vandini@unimore.it (E.V.); daniela.giuliani@unimore.it (D.G.); 3 International School of Advanced Studies, University of Camerino, 62032 Camerino, Italy

**Keywords:** Alzheimer’s disease, H_2_S-richwater, behavior, mouse model, neuroprotection

## Abstract

**Highlights:**

**What are the main findings?**

- Chronic H_2_S-rich water inhalation reduces early Aβ pathology and selectively modulates neuroimmune responses in 5XFAD mice.
- H_2_S-rich water exposure improves spatial search strategies specifically in female 5XFAD mice and increases total circulating H_2_S without overt toxicity.

**What are the implications of the main findings?**

- H_2_S-rich water inhalation may target multiple processes associated with early Aβ-driven pathology before overt cognitive impairment develops
- Non-invasive H_2_S delivery warrants further investigation as a potentially translatable approach for early intervention in AD.

**Abstract:**

Background: Alzheimer’s disease (AD) is a chronic neurodegenerative disorder characterized by progressive cognitive decline and behavioral impairments. Hydrogen sulfide (H_2_S) is an endogenous gasotransmitter that exerts pleiotropic neuromodulatory, anti-inflammatory, cytoprotective, and trophic effects within the central nervous system. Objectuves: This study investigated whether chronic inhalation of naturally H_2_S-rich sulphureous water, a non-invasive and clinically applicable intervention, exerts neuroprotective effects in 5XFAD mice, a transgenic model of severe AD pathology. Methods: Two-month-old male and female wild-type and 5XFAD mice underwent daily inhalation of saline or sulphureous water for eight weeks. Sulphureous water exposure was well tolerated and did not impair baseline learning or memory in either genotype. A detailed analysis of swim trajectories revealed selective enhancement of spatially accurate navigation strategies in sulphureous water-exposed 5XFAD female mice, with nearly a threefold increase in effective search behaviors, whereas male mice showed no treatment-specific effects. In addition, sulphureous water inhalation significantly reduced the number and area of 6E10^+^ and 11A1^+^ amyloid-β plaques in corticohippocampal regions and decreased cortical microglia number. Transcriptional profiling of cortical tissue revealed strong genotype-dependent upregulation of microglial, inflammatory, and stress-response genes. Sulphureous water exposure selectively modulated inflammatory and stress-response genes, including *Cd11b*, *Cst*, *Sod1*, *Tgfb1*, and *Hsp90a*, with distinct genotype-dependent interactions. Results: Overall, chronic inhalation of naturally H_2_S-rich sulphureous water during the prodromal phase of AD-like pathology mitigated early amyloid burden, reduced cortical microgliosis, and improved qualitative aspects of spatial navigation, particularly in female mice. Conclusions: These findings warrant further translational investigation of sulphureous water inhalation as a potential preventive or disease-modifying strategy for AD in humans.

## 1. Introduction

Alzheimer’s disease (AD), the predominant form of dementia, is a chronic and progressive neurodegenerative disorder marked by a gradual decline in cognitive and memory functions accompanied by accumulation of aggregated proteins and widespread neuroinflammation. The incidence and prevalence of AD continue to rise every year, and according to the 2021 World Alzheimer Report, more than 55 million people worldwide are currently living with this condition. This growing burden underscores the urgent need for therapeutic strategies capable of preventing, delaying, or attenuating disease progression [1].

Despite extensive research, no effective treatments are available to halt the progression of AD and most pharmacological interventions primarily offer symptomatic relief without modifying the underlying neurodegenerative processes, which include amyloid-β (Aβ) aggregation, tau pathology, synaptic dysfunction, and chronic neuroinflammation; in addition, novel therapies have mostly failed in clinical studies, except for the expensive monoclonal antibodies (mAbs), such as Aducanumab [2]. Among emerging Aβ-targeting mAbs, Lecanemab has recently been approved in the United States and parts of Asia for patients with mild cognitive impairment due to AD or mild AD dementia who carry only one or no copy of *apolipoprotein E ε4* gene. However, substantial concerns regarding its long-term efficacy and safety profile remain unsolved [3,4,5]. Hence, if on the one hand extensive research on innovative approaches for AD therapy is urgently required, on the other hand a comprehensive understanding of AD multifaceted pathogenesis remains crucial for developing novel disease-modifying interventions aimed at slowing down cognitive decline and improving the quality of life of patients and their families.

Increasing evidence indicates that oxidative stress plays a major role in AD pathophysiology, and disturbances in redox homeostasis are closely linked to neurotoxicity and neurodegeneration. Hydrogen sulfide (H_2_S), like nitric oxide and carbon monoxide, is a gasotransmitter with pleiotropic biological roles [6,7], including the regulation of redox balance. In addition, H_2_S contributes to protein sulfhydration, a post-translational modification involved in diverse cytoprotective biochemical pathways. If on one side, based on these premises, an increasing number of data support the beneficial effects of naturally sulphureous water for human health, on the other side H_2_S was once considered a toxic molecule. In fact, a biphasic effect has been demonstrated, exhibiting cytoprotective and beneficial actions at low concentrations [8] and cytotoxicity higher doses [9,10,11,12,13,14]. This dual nature underscores the importance of controlled and physiologically relevant delivery methods when exploring H_2_S-based therapeutic strategies.

Beyond its traditional use in medical hydrology for treating dermatological, respiratory, and neuromusculoskeletal conditions, topical and systemic applications of sulphureous water have demonstrated significant effects across a range of physiological and pathological contexts, including hypertension, atherosclerosis, ischemia/reperfusion injury, and chronic inflammatory diseases [15,16]. In the literature, most of these beneficial effects have been attributed to H_2_S released from sulfur-containing compounds or pharmacological donors such as sodium hydrosulfide (NaHS) [17,18,19].

In mammals, including humans, H_2_S is endogenously produced from L-cysteine metabolism through the action of cystathionine β-synthase (CBS), cystathionine γ-lyase (CSE), and cysteine aminotransferase (CAT). Endogenous H_2_S is also generated by the combined action of CAT, localized in the cytosol and 3-mercaptopyruvate sulfurtransferase (3-MST), present in the mitochondria. Of note, CSE-derived H_2_S is also a result of L-homocysteine metabolism [20]. In the central nervous system, glial CBS and neuronal 3-MST/CSE represent the primary enzymatic sources of H_2_S, contributing to its cell-specific signaling roles [21]. Notably, endogenous H_2_S levels decrease with ageing [22] and H_2_S synthesis is severely decreased in the brains of AD patients [23], while plasmatic H_2_S inversely correlates with AD severity [24]. Consistent with these findings, we and others have demonstrated that chronic systemic exposure to H_2_S or H_2_S-releasing compounds improves motor and cognitive decline in two rodent AD models—aged 3XTg mice and rats—accompanied by reductions in Aβ_1–42_ and phospho-tau levels [23,25,26].

To further investigate the potential therapeutic effects of sulphureous water on early AD-associated amyloidosis and inflammation, we employed the 5XFAD mouse model. These mice manifest an accelerated and severe AD-like pathology, with intracellular and extracellular Aβ accumulation evident as early as two months of age, followed by increasing neuroinflammation, cognitive impairment, and neurodegeneration at later stages [27]. In the present study, 5XFAD mice were chronically exposed to sulphureous water by inhalation (a non-invasive and clinically applicable intervention), and cognitive performance was assessed using the Morris Water Maze (MWM) test. Changes in key hallmarks of AD pathology—Aβ deposition and neuroinflammation—were examined through histological and molecular analyses, and serum H_2_S levels were measured. Chronic inhalation significantly reduced Aβ burden and early cortical microgliosis. Additionally, gene expression analysis revealed variations in immune surveillance- and amyloid clearance-related genes, while free H_2_S serum levels remained largely unchanged. These findings demonstrate that chronic sulphureous water inhalation modulates amyloid pathology, neuroinflammation, and cognitive function in a sex-dependent manner, supporting its potential as a safe, non-invasive intervention targeting early AD.

## 2. Materials and Methods

### 2.1. Animals

For this study, 2-month-old (at study onset) 5XFAD female (F) and male (M) mice (transgenic, Tg) and their wild-type (WT) littermates were used. 5XFAD mice co-overexpress a triple-mutant human *Amyloid precursor protein* (APP)—with Swedish (K670N, M671L), Florida (I716V), London (V717I) mutations—and a double-mutant human *Presenilin 1* (PS1) (M146L and L286V) transgenes under the transcriptional control of the neuron-specific *Thy-1* promoter [28]. Progenitors lacking the retinal degeneration allele *Pde6brd1* were purchased from Jackson Laboratories (Strain #034848-Bar Harbor, ME, USA). The 5XFAD line was maintained on its original hybrid B6SJL/J background by crossing hemizygous 5XFAD mice with hybrid B6SJL/J breeders. Mice were housed in air-conditioned colony rooms (temperature 21 ± 1 °C, humidity 60%) under a 12 h (hr) light/dark cycle. Food and water were available ad libitum. All experiments were carried out in strict accordance with the National Institutes of Health guidelines and were approved by the Committee on Animal Health and Care of Modena and Reggio Emilia University (protocol number: n°33220/2018-PR. Approved on 23 January 2018).

### 2.2. Experimental Groups and Treatment

WT and Tg mice were exposed to a daily session of inhalation of sulphureous water (thermal water with high H_2_S content; kindly provided by Salsomaggiore and Tabiano Thermae, Tabiano, Italy) or 0.9% NaCl solution (saline, sal) for 8 weeks. Sulphureous water, hereafter referred to as SPA, originates from Tabiano’s source Pergoli with the following characteristics: 125 mg/L H_2_S level (43 mg/L HS^-^ and 82 mg/L not ionized H_2_S), pH 6.6, 41 mOsm/L osmolarity [29]. SPA was administered by means of a Plexiglass inhalation chamber (15 × 8 × 5 cm) connected to an aerosol dispenser (AirCube, PIC). For each administration, mice were placed individually into the saturated chamber for 30–60 s. The individual exposure duration was dynamically adjusted according to each animal’s body weight and the dispenser flow rate (0.8 mL/min), using a physiological scaling factor of 0.2 mL of aerosol per mL of estimated mouse blood volume. This approach allowed us to target a nominal daily dose of approximately 2 mg/kg of total H_2_S across all animals (WT sal: *n* = 11 M, 9 F; WT SPA: *n* = 11 M, 10 F; Tg sal: *n* = 15 M, 11 F; Tg SPA: *n* = 11 M, 14 F). The experimental SPA dose of 2 mg/kg was selected based on the therapeutic dose currently used in humans for the inhalation treatment of respiratory tract inflammation [30].

### 2.3. Behavior Assessment

#### Morris Water Maze Test

Male and female mice were tested in the MWM, using a circular pool (1 m diameter) filled with opaque water (24 ± 1 °C), obtained by adding non-toxic white paint. During the learning phase, mice were allowed to swim for 90 s or until they mounted on a hidden circular white platform (11 cm diameter). Animals underwent 4 trials per day (starting from a different quadrant, with two different or no visual cue, at each trial) for 5 days with a 30 min (min) long inter-trial interval. For each trial, the latency to reach the platform was recorded and used to generate learning curves. Animals not reaching the platform were manually guided on it at the end of each trial, to ensure platform awareness. No visible trial was administered to the animals.

To assess reference memory at the end of the learning phase, 24 h after the last acquisition day, a Probe test was conducted. The platform was removed and the animals were allowed to swim freely for 60 s. The latency to reach the platform location, total entries in the platform area and the percentage (%) of time spent in target quadrant (platform area preference) were quantified. Additionally, navigation strategies were analyzed according to validated protocols [31,32]. Briefly, individual swim paths (track plots) generated by the ANY-maze software during the Probe test were visually classified into distinct search strategies [31,33], as follows: *spatial accurate strategies* included direct or slightly deviated paths oriented toward the platform location; *spatial inaccurate strategies* involved focused searching behaviors confined either to the correct platform quadrant or to an incorrect one; *non-cognitive strategies* showed no efficient spatial orientation or consisted of completely unselective random paths. Finally, *no target behaviors* were defined as those where the animal showed no spatial orientation and completely failed to locate the platform area during the test.

All behavioral testing was performed by an operator blind to treatment to avoid any bias. Experiments were conducted in a sound-proof room and behavior was recorded and automatically analyzed with ANY-maze video tracking system (Stoelting, 7.67).

### 2.4. Animal Sacrifice and Ex Vivo Analysis

At the end of the behavioral testing, mice were deeply anesthetized with isoflurane and sacrificed by intracardiac infusion of 50 mL of 0.9% NaCl and 70 mL of cold 4% paraformaldehyde in phosphate-buffered saline (PBS) using a peristaltic pump (10 mL/min flowrate) following blood draw. Brains were collected, post-fixed for 24 h in the same fixative solution, transferred to 30% sucrose in PBS for cryoprotection for 2 days, and then rapidly frozen on dry ice. Samples were cut at a cryostat to obtain 20 µm coronal sections and processed for histological analyses according to established protocols [27,31].

### 2.5. Immunohistochemical Analysis

Immunohistochemical (IHC) analyses were performed to test cell-specific AD-linked alterations using the following specific antibodies (Abs) against amyloid plaques and microglial cells: mouse anti-human Aβ/APP 6E10 (epitope human Aβ1–16, 1:500, Signet, Dedham, MA, USA, #9300-10), mouse anti-human Aβ 11A1 (epitope synthetic peptide of E22P-Aβ 10–35 part, 1:200, Tecan, Männedorf, Switzerland, #10379), rabbit anti-ionized calcium-binding adapter molecule 1 (Iba1, 1:1000, Wako, Osaka, Japan, #019-19741). A pre-treatment with formic acid was performed for anti-Aβ Abs. Vectastain ABC-HRP (Vector Laboratories, Burlingame, CA, USA, #PK-4002; #PK-4001) kit was used for peroxidase diaminobenzidine staining; slices were placed on gelatinized glass slides, dehydrated and mounted with Eukitt^®^ (O. Kindler GmbH, Freiburg, Germany) mounting medium. For concurrent analysis of microglia and plaques, Iba1-labelled slides were co-stained with Congo Red (Sigma, St. Louis, MO, USA, #6277) according to established protocols [27,31].

### 2.6. Image Analysis

Immunolabelled brain slices were visualized and photographed with a Nikon Eclipse CiL microscope through a Nikon DS-Fi3 camera under constant light conditions. Shading correction was performed before image acquisition to minimize light-dependent irregularities of the microscopic field. All evaluations were carried out blindly on coded slides by at least two experimenters. Analyses were performed on cerebral cortex overlying the dorsal hippocampus (CTX) and the dentate gyrus (DG) hippocampal subfield (Bregma between −1.46 and −1.58 mm from Paxinos and Franklin) in at least 3 slices/area/mouse.

Aβ 6E10- and 11A1-immunoreactive plaques were manually selected with a 10X objective and morphometric parameters such as plaque number in 500,000 µm^2^ as well as single plaque area were measured through a Nikon NIS-Elements D software (BR 6.10.01). For each animal, the area of individual 6E10^+^ and 11A1^+^ plaques was measured within the analyzed CTX and DG regions, and the mean plaque area was calculated and used as the experimental unit for statistical analysis. For Iba1 analysis, images were acquired using a 10× objective, converted to 32 bit grayscale and binarized applying the following thresholds: –20 grayscale units from mean gray peak (background) for CTX and –15 for DG hilus; then, % of immunoreactive positive area, i.e., the area covered by pixels with gray value above the threshold, was automatically recorded by means of analyze particle function of ImageJ software (1.54p). Additionally, Iba1^+^ cell count was performed manually, within the area of interest (500,000 µm^2^) by using a 10× objective.

Iba1^+^/Congo Red co-staining analysis was performed on the CTX; images were acquired at 20× magnification and the number of Iba1^+^ clusters around plaques was manually counted and normalized over 6E10^+^ plaque number. Additionally, the area of each cluster was measured using Nikon NIS-Elements D software.

### 2.7. Detection of Serum H_2_S Levels Through HPLC-FLD Method

Blood was collected before intracardiac perfusion into serum clot activator tubes and kept at room temperature for 30 min, then centrifuged at 1200× *g* for 15 min. Serum was isolated and stored at −80 °C until use. For the quantification of free serum H_2_S, a method based on the use of the fluorescent reagent monobromobimane was used to bind H_2_S with formation of the sulfide dibimane (SDB) derivative, which was subsequently determined by reversed-phase High-Performance Liquid Chromatography (HPLC) and fluorescence detection (FLD).

Separation and quantification of SDB was carried out using Agilent HPLC-FLD instrumentation: Agilent 1100 Series Mobile Controller, Agilent 1100 Series Autosampler, Agilent 1100 Series HPLC Pump, Agilent 1200 Series Vacuum Degasser, Agilent 1200 Series Diode Array Detector, Agilent 1200 fluorescence detector, Agilent HPLC 2D ChemStation SW Licence 32 bit.

A C18 column (C18 Agilent Zorbax Eclipse XDB—C18 4.6 × 250 mm, 5 µm) with pre-column, a mobile phase A (water with 0.1% trifluoroacetic acid)/B (acetonitrile with 0.1% trifluoroacetic acid) and gradient elution was used for chromatographic separation. Detection was performed by recording the fluorescence signal with λ_Ex_ = 390 nm and λ_Em_ = 475 nm. After derivatization, the samples were stored at room temperature during the analysis sequence.

### 2.8. Total RNA Extraction, Reverse Transcription, and Real-Time Polymerase Chain Reaction

A separate group of mice (*n* = 3 animals/sex/group) was sacrificed under deep anesthesia through intracardiac injection of cold PBS. Brains were rapidly dissected, and CTX was collected and lysed by mechanical disruption in Qiazol reagent (Qiagen, Hilden, Germany). Tissue homogenization and RNA extraction were performed according to the procedure provided by the manufacturer (RNeasy Plus Mini Kit, Qiagen) and processed for Real-Time quantitative PCR (RT-qPCR) as previously described [34,35].

Isolated mRNA was reverse transcribed into 30 ng/μl cDNA using random hexamers and M-MLV Reverse Transcriptase (Promega Corporation, Madison, WI, USA) according to the manufacturer’s instructions. Samples were heated at 70 °C for 5 min to eliminate any secondary structures, then incubated at 23 °C for 10 min, 1 h at 37 °C, and 5 min at 95 °C before being chilled at 4 °C using a thermocycler T Gradient (SimpliAmp, Applied Biosystem, Waltham, MA, USA).

The amount of cDNA was quantified with iTaq Universal SYBR Green Supermix (Bio-Rad, Hercules, CA, USA) using a Bio-Rad RT-PCR iCycler. Each PCR reaction was performed in duplicate using 300 nM of each primer (Table 1), 10 μL of iTaq Universal SYBR Green Supermix (Bio-Rad), 1 μL cDNA and nuclease-free water with the following cycling parameters: 2 min at 95 °C and 40 cycles of 5 s at 95 °C and 30 s at 60 °C, followed by 5 s at 95 °C and from 65 to 95 °C with 0.5 °C increment for melting curve analysis.

### 2.9. Statistical Analysis

Data are shown as mean ± standard error of the mean (SEM) or standard deviation (SD). For behavioral analysis, group differences were analyzed by two-way analysis of variance (ANOVA) or repeated measure ANOVA, as appropriate. For conventional MWM parameters, genotype and treatment were included as factors and sex as covariate. Escape latency during the acquisition phase was analyzed by repeated-measures ANOVA, with training day as the within-subject factor. Probe trial parameters, including the number of entries into the platform area, latency to reach the former platform location, and percentage of time spent in the target quadrant, were analyzed by two-way ANOVA.

Search strategies were analyzed separately in female and male mice, and differences in the distribution of spatially accurate and non-cognitive strategies among experimental groups were assessed using Fisher’s exact test.

Mean plaque area was compared between treatment groups by univariate ANOVA, with treatment as the main factor and sex as covariate. For plaque-size distribution analysis, individual plaques were additionally classified into four predefined size categories [31], and the relative proportion of plaques within each size category was compared between treatment groups using Fisher’s exact test.

For gene expression analysis, mRNA levels of each target gene were normalized over the mean Cycle Threshold (Ct) of *Gapdh* and *Rps29* reference housekeeping genes [35]. Quantitative changes were evaluated using the comparative ΔΔCt method, with the average expression levels of WT sal mice serving as calibrators. Statistical comparisons were performed using two-way ANOVA, with genotype and treatment as factors and sex as covariate.

All statistical analyses were performed through SPSS software (version 26), with *p* < 0.05 as the level for a significant difference (*p* < 0.05 *; *p* ≤ 0.01 **; *p* ≤ 0.001 ***) and 0.05 ≤ *p* ≤ 0.10 as trend for a significant difference (#).

## 3. Results

### 3.1. Chronic SPA Exposure Improves Spatial Navigation in Female 5XFAD Mice

**Figure 1:**
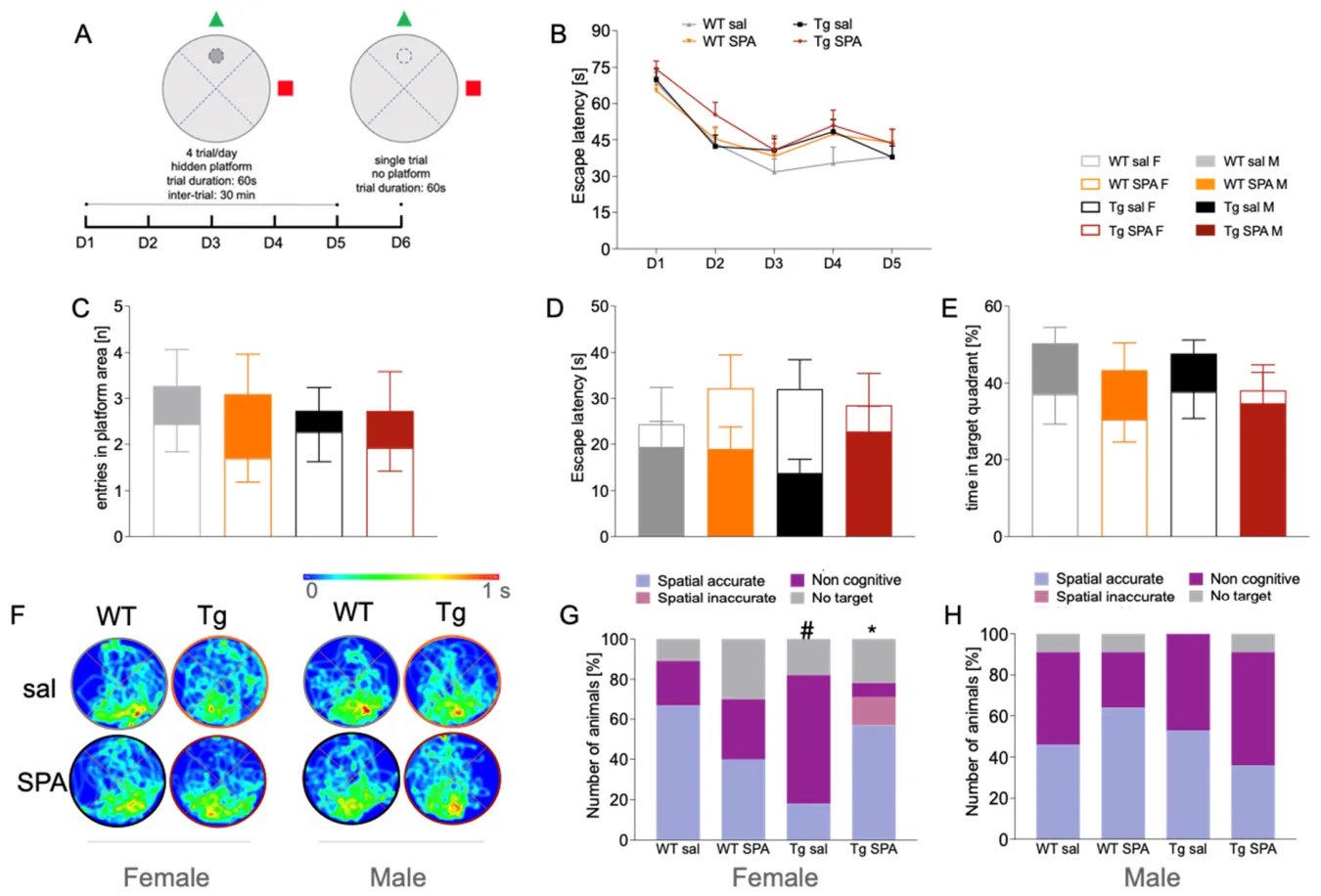
**Chronic SPA exposure did not affect learning and memory in young WT and Tg mice but improved platform spatial search strategies in female Tg mice**. (**A**) Experimental workflow illustrating the MWM protocol. (**B**) Learning curve of experimental mice showing the time needed to find the platform (escape latency averaged over 4 trials/day) over the 5 testing days (D1–5). No significant differences were observed between SPA- and sal-treated groups (Repeated measures ANOVA with training day, genotype and treatment as factors; sex as covariate: Treatment F (1, 87) = 1.012, *p* = 0.317; Genotype F (1, 87) = 1.146, *p* = 0.287; Sex F (1, 87) = 3.721, *p* = 0.057; Genotype × Treatment F (1, 87) = 0.001, *p* = 0.973; Training day F (4, 348) = 10.757, *p* < 0.001). Data showed as mean ± SEM. (**C**) Number of entries in the platform area during the Probe test showed no difference between experimental groups (Two-way ANOVA with genotype and treatment as factors; sex as covariate: Treatment F (1, 91) = 0.399, *p* = 0.529; Genotype F (1, 91) = 0.245, *p* = 0.622; Sex F (1, 91) = 3.295, *p* = 0.073 (#); Genotype × Treatment F (1, 91) = 0.108, *p* = 0.743). Data showed as mean ± SEM. (**D**) Latency to reach the platform location during Probe test was not different between groups (Two-way ANOVA with genotype and treatment as factors; sex as covariate: Treatment F (1, 91) = 0.583, *p* = 0.447; Genotype F (1, 91) = 0.000, *p* = 0.995; Sex F (1, 91) = 6.685, *p* = 0.011 (*); Genotype × Treatment F (1, 91) = 0.001, *p* = 0.973). Data showed as mean ± SEM. (**E**) % of time spent in the target quadrant during the Probe test was comparable between groups (Two-way ANOVA with genotype and treatment as factors; sex as covariate: Treatment F (1, 87) = 1.201, *p* = 0.276; Genotype F (1, 87) = 0.097, *p* = 0.756; Sex F (1, 87) = 0.295, *p* = 0.588; Genotype × Treatment F (1, 87) = 0.127, *p* = 0.722).) Data showed as mean ± SEM. (**F**) Occupancy plots for female (left) and male (right) grouped data during the Probe test was not different between groups; the color scale represents the mean time spent in each location over the 60 s trial, ranging from blue (low occupancy, 0 s) to red (high occupancy, 1 s). (**G**,**H**) Analysis of platform search strategy during the Probe test in female (**G**) and male (**H**) mice. SPA treatment significantly increased the use of spatial accurate strategies in female Tg SPA mice compared to Tg sal mice (Fisher’s exact test, **Female:** all groups, *p* = 0.0935 (#); WT sal vs. WT SPA, *p* = 0.590; WT sal vs. Tg sal, *p* = 0.088 (#); Tg sal vs. Tg SPA, *p* = 0.015 (*); WT SPA vs. Tg sal, *p* = 0.339; WT SPA vs. Tg SPA, *p* = 0.380. **Male**: all groups, *p* = 0.748). **Colour legend**: light purple, spatial accurate; pink, spatial inaccurate; purple, non-cognitive; grey, no target. **Symbol legend**: WT sal: *n* = 11 M (filled grey bar), 9 F (empty grey bar); WT SPA: *n* = 11 M (filled orange bar), 10 F (empty orange bar); Tg sal: *n* = 15 M (filled black bar), 11 F (empty black bar); Tg SPA: *n* = 11 M (filled burgundy bar), 14 F (empty burgundy bar). **Abbreviations**: D: day, F: female, M: male, sal: saline, SPA: sulphureous water, Tg: transgenic, WT: wild type.

It is known that H_2_S may exert beneficial or detrimental effects at low or high doses, respectively [8,11,12,36,37,38,39]. So, we first investigated whether the present chronic H_2_S inhalation protocol exerted beneficial or adverse effects on cognitive function, when administered during the prodromal phase of AD-like pathology. Spatial learning and memory were evaluated using the MWM test, a widely established behavioral test for hippocampus-dependent cognition [31]. The experimental timeline is represented in Figure 1A.

During the learning phase of the MWM test, mice progressively learned the location of the hidden platform, with no significant differences in escape latency among the experimental groups (Figure 1B). On day 6 (D6), the platform was removed to evaluate spatial reference memory in a probe test. The number of platform location crossings (Figure 1C) and the percentage of time spent in the target quadrant (Figure 1E) did not differ significantly between groups, regardless of sex and treatment. Similarly, the treatment did not affect the latency to reach the former platform location (Figure 1D), although a significant main effect of sex was detected (*p* = 0.011). Additionally, the average swimming speed and the total swimming distance travelled were comparable across all experimental groups (not shown), indicating that swimming performance was not affected.

Interestingly, the significant effect of sex observed for the latency to reach the former platform location suggested potential sex-related differences in spatial navigation that might not be captured by conventional MWM parameters. Together with the well-known sexual dimorphism in spatial navigation, this finding guided our sex-stratified analysis of swimming trajectories and search strategies. In fact, the qualitative analysis of search strategies revealed different patterns between sexes. Female Tg sal mice relied more heavily on non-cognitive strategies than WT sal mice (64% vs. 22%, respectively). Chronic SPA exposure significantly improved the use of spatially accurate strategies in female Tg mice, with the proportion of accurate strategies increasing from 18% in Tg sal to 57% in Tg SPA mice (Figure 1G). In contrast, male mice, regardless of genotype or treatment, predominantly used spatially accurate strategies to locate the former platform location during the probe test (Figure 1H). Statistical analyses for all comparisons are reported in Figure 1 legend.

Overall, the present data show that learning and memory performance in the MWM test did not differ between WT and Tg mice during the early stage of AD pathology development (4 months of age). Under this condition, H_2_S inhalation protocol did not affect cognitive performance in either genotype. However, a more detailed analysis revealed that H_2_S treatment significantly ameliorated the searching strategies of female Tg mice, baseline-inclined to rely on non-cognitive strategies more than their male counterparts.

### 3.2. Chronic SPA Exposure Reduces Amyloid Pathology and Alleviates Neuroinflammation in 5XFAD Mice

#### 3.2.1. Amyloid Plaques

**Figure 2:**
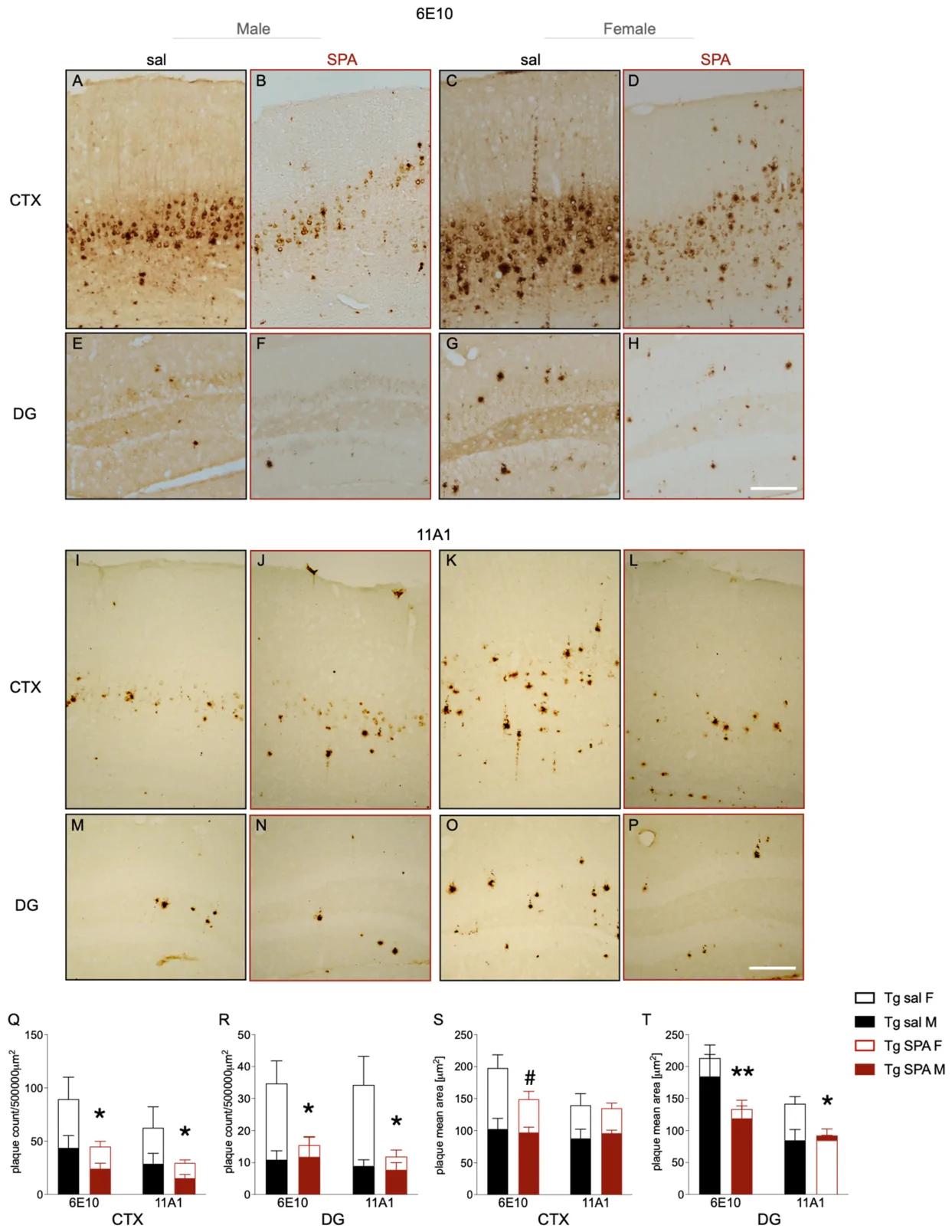
**Chronic SPA exposure significantly reduced amyloid plaque deposition in the CTX and DG of male and female 5XFAD Tg mice**. (**A**–**P**). Magnified details of representative images of 6E10^+^ and 11A1^+^ plaques in the CTX (**A**–**D** and **I**–**L**) and hippocampal DG (**E**–**H** and **M**–**P**) of male and female Tg sal- and SPA-exposed mice. Scale bar = 100 μm. (**Q**–**T**) 6E10^+^ and 11A1^+^ plaque count (**Q**,**R**) and mean area (**S**,**T**) of Tg sal- and SPA-exposed mice. Data are shown as mean ± SEM and compared by univariate ANOVA, with treatment as factor and sex as covariate (**6E10**: **Plaque count**: **CTX**: Treatment F (1, 19) = 7.538, *p* = 0.014 (*); Sex (covariate) F (1, 19) = 7.106, *p* = 0.016 (*); **DG**: Treatment F (1, 19) = 5.012, *p* = 0 039 (*); Sex (covariate) F (1, 19) = 7.920, *p* = 0.012 (*). **Plaque mean area**: **CTX**: Treatment F (1, 19) = 3.555, *p* = 0.077 (#); Sex (covariate) F (1, 19) = 19.415, *p* < 0.001, **DG**: Treatment F (1, 19) = 10.460, *p* = 0.005 (**); Sex (covariate) F (1, 17) = 0.914, *p* = 0.353; **11A1**: **Plaque count**: **CTX**: Treatment F (1, 19) = 4.986, *p* = 0.039 (*); Sex (covariate) F (1, 19) = 4.649, *p* = 0.046 (*); **DG**: Treatment F (1, 19) = 6.779, *p* = 0.019 (*); Sex (covariate) F (1, 19) = 8.035, *p* = 0.011 (*); **Plaque mean area**: **CTX**: Treatment F (1, 19) = 0.001, *p* = 0.980; Sex (covariate) F (1, 19) = 11.963, *p* = 0.003 (**); **DG**: Treatment F (1, 19) = 5.117, *p* = 0.037 (*); Sex (covariate) F (1, 19) = 3.578, *p* = 0.069 (#)). **Symbol legend**: Tg sal: *n* = 5 M (filled black bar), 5 F (empty black bar); Tg SPA: *n* = 3 M (filled burgundy bar), 7 F (empty burgundy bar). **Abbreviations**: CTX: cerebral cortex; DG: dentate gyrus; M: male; F: female; sal: saline; SPA: sulphureous water; Tg: transgenic.

The effects of SPA exposure on amyloidosis were assessed through IHC in coronal brain slices using 6E10- and 11A1-specific anti-Aβ Abs. Representative images of 6E10^+^ and 11A1^+^ plaques in the CTX and DG are shown in Figure 2A–H and Figure 2I–P, respectively. Quantitative analysis revealed that chronic SPA exposure significantly reduced amyloid burden in Tg mice. Specifically, SPA treatment decreased the number of amyloid plaques in both the CTX (**6E10**:— − 42%, *p* = 0.014; **11A1**: − 45%, *p* = 0.039) and DG (**6E10**: −37%, *p* = 0.039; **11A1**: −51%, *p* = 0.019) (Figure 2Q,R). A reduction in mean plaque area was also observed, reaching significance in the DG (**6E10**: − 35%, *p* = 0.005; **11A1**: − 23%, *p* = 0.037), and showing a trend toward a significant effect in the CTX (**6E10**: − 11%, *p* = 0.077; **11A1**: + 8%, *p* = 0.980) (Figure 2S,T). Together, these findings demonstrate that chronic SPA exposure attenuated amyloid pathology in both cortical and hippocampal regions of Tg mice, with a particularly pronounced reduction in the area of Aβ^+^ plaques in the DG.

To further characterize the effect of SPA on plaque morphology, amyloid plaques in the CTX and DG of mice were stratified by size into four categories, as previously described [31]. Consistent with the overall reduction in amyloid burden (see above), the absolute number of Aβ^+^ plaques for each size range was reduced across all size categories (not shown). However, the relative distribution of plaques among different size categories remained unchanged following SPA exposure. Specifically, the proportion of plaques across different size ranges was maintained in both the CTX and the DG of SPA-treated Tg mice for both 6E10^+^ and 11A1^+^ populations (Fisher’s exact test: **6E10**: DG: *p* = 0.956, CTX: *p* = 0.631; **11A1**: DG: *p* > 0.999, CTX: *p* = 0.984). These findings indicate that SPA reduced overall plaque burden without preferentially affecting plaques of a specific size range, suggesting a proportional reduction across the entire plaque population (Supplementary Figure S1) [27]. Detailed statistical analyses are reported in Supplementary Figure S1 legend.

#### 3.2.2. Ionized Calcium-Binding Adapter Molecule 1 Immunoreactivity

Recently, microglial processes have been recognized as crucial contributors to the initiation and progression of AD pathology [40]. To evaluate the effects of SPA on microglia, we examined Iba1 immunoreactivity (IR), a marker of microglial cells, in the four experimental groups. Given that the CTX and DG are among the first affected regions during the prodromal stages of AD-like pathology in 5XFAD mice, Iba1 IR was quantified specifically in these brain areas [27,41].

**Figure 3:**
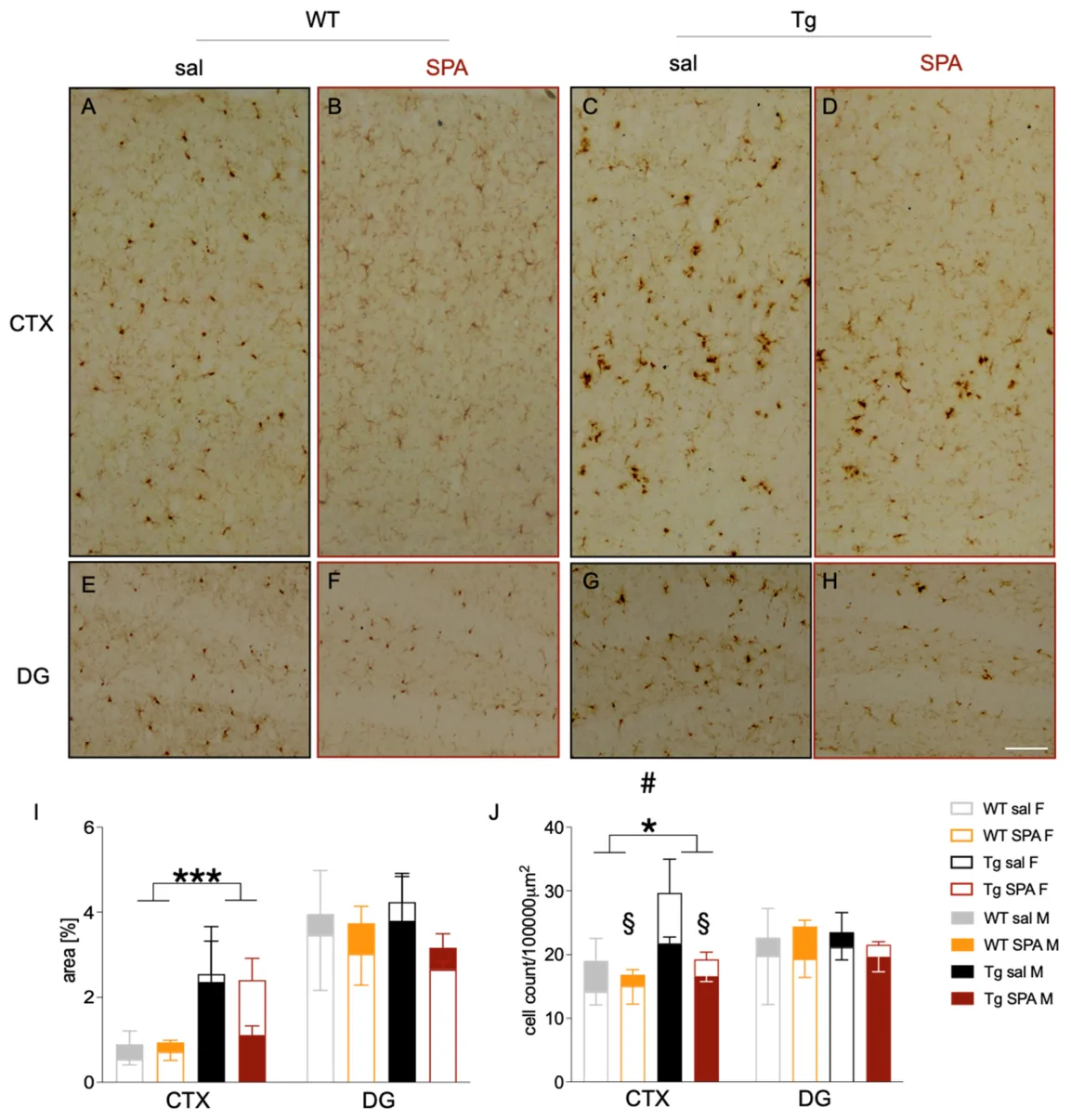
**Quantitative analysis of Iba1 IR in CTX and DG of WT and Tg mice chronically exposed to sal or SPA**. (**A**–**H**) Magnified details of representative images of Iba1^+^ staining in the CTX (**A**–**D**) and DG (**E**–**H**) of WT (**A**,**B**,**E**,**F**) and Tg (**C**,**D**,**G**,**H**) mice exposed to sal (**A**,**C**,**E**,**G**) or SPA (**B**,**D**,**F**,**H**). Scale bar  =  200 μm. (**I**,**J**) Quantitative analysis related to the IR area (**I**) and the number (**J**) of Iba1^+^ cells in CTX and DG of experimental mice. Data are shown as mean ± SEM and compared by repeated measure ANOVA (**CTX: Area:** Treatment F (1, 23) = 0.695, *p* = 0.413; Genotype F (1, 23) = 14.384, *p* < 0.001 (***); Sex (covariate) F (1, 23) = 0.188, *p* = 0.669; Genotype × Treatment F (1, 23) = 1.314, *p* = 0.264; **Count**: Treatment F (1, 23) = 4.311, *p* = 0.049 (§); Genotype F (1, 23) = 7.504, *p* = 0.012 (*); Sex (covariate) F (1, 23) = 0.035, *p* = 0.853; Genotype × Treatment F (1, 23) = 3.098, *p* = 0.092 (**#**). **DG: Area**: Treatment F (1, 23) = 1.561, *p* = 0.224; Genotype F (1, 23) = 0.019, *p* = 0.891; Sex (covariate) F (1, 23) = 0.410, *p* = 0.528; Genotype × Treatment F (1, 23) = 0.457, *p* = 0.506; **Count**: Treatment F (1, 23) = 0.040, *p* = 0.843; Genotype F (1, 23) = 0.001, *p* = 0.977; Sex (covariate) F (1, 23) = 0.740, *p* = 0.398; Genotype × Treatment F (1, 23) = 0.185, *p* = 0.671). **Symbol legend**: WT sal: *n* = 4 M (filled grey bar), 4 F (empty grey bar); WT SPA: *n* = 4 M (filled orange bar), 4 F (empty orange bar); Tg sal: *n* = 3 M (filled black bar), 3 F (empty black bar); Tg SPA: *n* = 3 M (filled burgundy bar), 3 F (empty burgundy bar). **Abbreviations**: CTX: cerebral cortex, DG: dentate gyrus, M: male, F: female, sal: saline, SPA: sulphureous water, Tg: transgenic, WT: wild type.

In the CTX, the quantitative analysis revealed a significant genotype-dependent effect, with Tg mice displaying increased Iba1 IR compared with WT mice, consistent with the well-established microgliosis associated with amyloid pathology [27]. Even if this increase in cortical Iba1 IR (Figure 3A–D) was not significantly attenuated following chronic SPA exposure (Figure 3I), SPA treatment significantly reduced the number of Iba1^+^ cells (Figure 3J) in Tg mice compared to their sal-treated counterparts (+155% Tg sal and +108% Tg SPA vs. Wt sal). Of note, no significant differences in mean microglial soma area were detected among the experimental groups (Sex: F (1, 27) = 0.075, *p* = 0.787; Treatment: F (1, 27) = 1.164, *p* = 0.292; Genotype: F (1, 27) = 0.681, *p* = 0.418; Genotype × Treatment: F (1, 27) = 1.055, *p* = 0.312) (Supplementary Figure S2A). Thus, SPA selectively reduced the number of cortical Iba^+^ cells without affecting overall Iba1 IR or microglial soma size at this disease stage.

In contrast, analysis of the DG (Figure 3E–H) revealed no significant effects of AD-like pathology or SPA treatment, as neither Iba1 IR nor the number of Iba1^+^ cells differed between WT and Tg groups (Figure 3I,J). Similarly, microglial soma mean area was preserved across experimental groups, indicating no detectable morphological changes in DG microglia at this stage of pathology (Supplementary Figure S2A).

To evaluate whether SPA exposure influenced microglial recruitment to amyloid plaques, the number and area of Congo Red/Iba1^+^ plaque-associated microglial clusters were quantified. No significant differences were detected in either the number of clusters (Sex: F (1, 11) = 0.025, *p* = 0.878; Treatment: F (1, 11) = 0.954, *p* = 0.354) or the mean cluster area (Sex: F (1, 11) = 0.252, *p* = 0.627; Treatment: F (1, 11) = 1.495, *p* = 0.253) between SPA- and sal-treated Tg mice (Supplementary Figure S2B). Thus, although SPA reduced overall plaque burden and cortical microgliosis, it did not significantly affect microglial clustering at this stage of the pathology. Detailed statistical analysis is reported in Supplementary Figure S2 Legend.

### 3.3. Toxicity

No signs of toxicity were observed throughout the 2-month SPA treatment, including ruffled fur, diarrhea, lethargy, aggressiveness, hypothermia, or alterations in spontaneous locomotor activity and grooming (data not shown).

### 3.4. H_2_S Serum Concentrations

Previous studies have reported that brain H_2_S synthesis is markedly decreased in AD patients, and that serum H_2_S levels correlate with disease severity both in patients and in AD mouse models [42,43].

**Figure 4:**
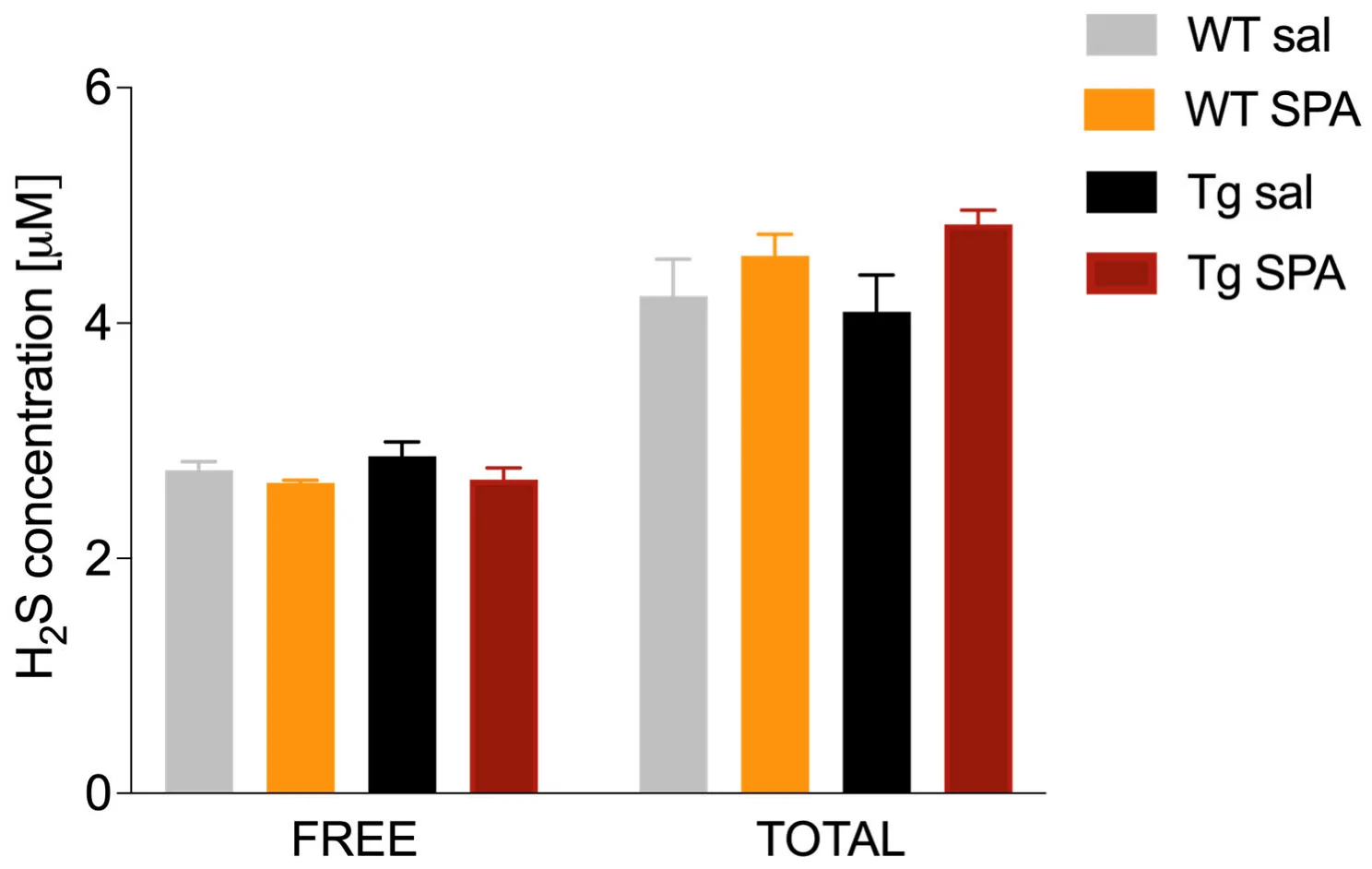
**H_2_S serum levels of WT and Tg mice after 2 months of sal or SPA treatment**. Total H_2_S concentration (µM) was consistently higher than free H_2_S in all groups (significant Form parameter) and significantly augmented in SPA-exposed mice, irrespective of the genotype (significant Form × Treatment interaction). Data are shown as mean ± SEM and compared by repeated measure ANOVA, with genotype and treatment as factors. Treatment: F (1, 15) = 1.442, *p* = 0.248; Genotype: F (1, 15) = 0.192, *p* = 0.668; Genotype × Treatment: F (1, 15) = 0.0.230, *p* = 0.639; H_2_S Form: F (1, 15) = 210.184, *p* < 0.001; H_2_S Form × Treatment: F (1, 15) = 8.762, *p* = 0.010; H_2_S Form × Genotype: F (1, 15) = 0.001, *p* = 0.979; H_2_S Form × Treatment × Genotype: F (1, 15) = 1.108, *p* = 0.309. **Symbol legend**: WT sal: *n* = 4 (grey bar); WT SPA: *n* = 4 (filled orange bar); Tg sal: *n* = 6 (black bar); Tg SPA: *n* = 5 (burgundy bar). **Abbreviations**: sal: saline, SPA: sulphureous water, Tg: transgenic, WT: wild type.

Serum analysis performed after 8 weeks of treatment indicated that total H_2_S concentration was consistently higher than free H_2_S concentrations across all experimental groups. Moreover, a significant H_2_S form × treatment interaction was detected, irrespective of the genotype indicating that SPA treatment specifically increases total H_2_S form in both genotypes (Figure 4).

### 3.5. Gene Expression Analysis on Cortices of Mice Sacrificed After Sal or SPA Exposure

Sal and SPA-exposed mice of both sexes were sacrificed after 8 weeks of treatment to assess long-term effects on gene expression. Total RNA was extracted from dissected CTX of WT and 5XFAD Tg mice, reverse transcribed and analyzed by RT-qPCR. A summary of the results is provided in Table 2. The gene panel included microglial markers (*Cd11b*, *Cst*), as well as mediators and effectors of inflammatory- and apoptotic- responses (e.g., *Ccl3*, *Ccl6*. *Hmgb1*, *Il1b*, *Il4*, *Il6*, *Il10*, *Sod1*, *Tgfb1*, *Tnfa*, *Hsp90a*, *Hif1a*).

RT-qPCR analysis revealed a strong effect of genotype across multiple inflammatory and stress-response genes. *Cd11b*, *Cst*, *Ccl3*, *Ccl6*, *Hmgb1*, *Il1b*, *Sod1* and *Tgfb1* showed robust main effects of genotype, with Tg mice consistently exhibiting higher expression levels than WT mice irrespective of treatment (all *p* < 0.001). *Tnfa*, and *Hsp90a* also showed a trend toward a higher expression in Tg animals, although these differences did not reach significance (*p* > 0.05).

The effects of SPA were more selective, reaching statistical significance only for *Cst*, *Sod1*, *Tgfb1*, and *Hsp90a* (*p* < 0.05). Significant genotype × treatment interaction was detected for, *Cst*, *Tgfb1*, and *Hsp90a* (*p* < 0.05), indicating that SPA treatment differentially altered these transcripts in WT and Tg groups. *Cd11b*, *Ccl3* and *Ccl6* showed similar patterns, although the interaction did not reach statistical significance.

Sex effects were detected for *Hsp90*, *Il1b*, *Hmgb1*, *Tnfa*, and *Hif1a*. No significant sex × treatment interactions were observed for these genes, with the exception of *Hsp90*, which showed a significant interaction between sex and treatment. In contrast, *Il4*, *Il6* and *Il10*, were not significantly affected by sex, genotype, treatment, or their interactions.

Overall, these findings identify genotype as the major determinant of cortical transcriptional changes, whereas SPA treatment selectively modulated specific components of the inflammatory and stress-response pathways, particularly through genotype-dependent effects in Tg mice.

## 4. Discussion

Recent evidence suggests that altered communication between neural cells arises before the clinical onset of AD, and the gaseous transmitter H_2_S is increasingly recognized as a critical mediator of these early alterations. Given its pleiotropic regulatory roles in neurotransmission, inflammation, apoptosis, bronchodilation, autophagy, and oxidative stress, changes in H_2_S metabolism may have widespread consequences for neuronal and glial function.

Multiple lines of preclinical and clinical data now support a role for altered H_2_S signaling in AD. Endogenous levels of H_2_S are significantly diminished in the brain of mouse models of AD (e.g., APP/PS1, 3XTg) [42,44], and similar deficits have been observed in human AD; in fact, both brain tissue and plasma H_2_S are reduced in AD patients [24,45], and plasma H_2_S level correlates inversely with disease severity [46]. However, these alterations may depend on the biological compartment, experimental model, and stage of disease investigated. Mechanistically, CSE, the principal H_2_S-producing enzyme in neurons, is markedly downregulated in AD. For example, postmortem cortex samples from AD brains show about 50% decrease in CSE expression, similar to that reported in 3XTg mice [23,47]. This reduction has functional consequences, as inadequate H_2_S production decreases protein sulfhydration—particularly of kinases such as GSK3β—thereby facilitating tau hyperphosphorylation and synaptic dysfunction [23]. More broadly, protein persulfidation represents an important H_2_S-dependent post-translational modification through which H_2_S can regulate protein activity, stability, and cellular signaling. Alterations in this process may therefore influence multiple pathways involved in AD-related proteostasis, potentially linking impaired H_2_S signaling not only to amyloid pathology but also to other proteinopathies and disease-associated post-translational modifications. Thus, modulation of protein persulfidation represents a plausible additional mechanism through which restoration of H_2_S signaling may exert neuroprotective effects. However, protein persulfidation and disease-associated post-translational modifications of tau, TDP-43 or other aggregation-prone proteins were not directly assessed in the present study; therefore, their contribution to the effects of SPA remains to be established.

Importantly, restoration of H_2_S signaling has demonstrated protective effects in both in vitro and in vivo AD models. Slow-releasing H_2_S donors, such as NaHS and GYY4137, rescue cognitive deficits, attenuate amyloid and tau pathology, and reduce neuroinflammation through both sulfhydration-dependent and independent mechanisms.

Previous in vivo studies in fact reported that NaHS treatment attenuates spatial memory impairment and decreases hippocampal neuroinflammation in 3XTg mice and rat models of AD [25,48,49]. Similarly, in vitro studies demonstrated that NaHS reduces neuroinflammation in Aβ-activated microglial cells and protects PC12 cells from Aβ-induced toxicity [50,51]. Other H_2_S-releasing compounds, including natural products and plant-based foods such as garlic, have also shown efficacy in preclinical AD models. Collectively, these findings provide the rationale for investigating whether restoration or supplementation of H_2_S signaling may modulate early AD-related pathological processes.

Interestingly, natural SPA waters are already recognized as helpful therapeutic approaches to improve the quality of life of patients belonging to several clinical settings, such as osteoarthritis [52,53], multiple respiratory diseases like asthma, chronic obstructive pulmonary disease [54] and otitis media [55]. This non-invasive approach provides exposure to physiologically tolerated levels of H_2_S and is easily accessible and already used in different clinical settings, supporting the rationale for investigating its potential translational applicability to AD-related pathological processes.

In this study, we reported the effects of chronic inhalator exposure to H_2_S-rich SPA water during the prodromal phase of AD-like pathology development in young male and female 5XFAD mice, a Tg mouse model of AD characterized by early Aβ accumulation. In 5XFAD mice, intracellular and extracellular Aβ begin to accumulate at 2 months of age and rapidly increase with age, along with inflammation, oxidative stress and neuronal loss, accompanied by learning and memory impairments [27]. Our results demonstrate that H_2_S administered via SPA water inhalation is well tolerated and does not interfere with baseline spatial learning or memory in young WT or Tg mice. Importantly, the absence of overt toxicity, together with the lack of effects on locomotor activity or baseline cognitive performance, supports the safety of chronic SPA inhalation and its suitability for long-term interventions under the experimental conditions used here. Moreover, chronic SPA exposure significantly increased total circulating H_2_S levels irrespective of genotype, while free serum H_2_S remained unchanged, demonstrating a measurable systemic effect of the inhalation protocol on the circulating H_2_S pool. Notably, under our experimental conditions, no decreases in basal circulating H_2_S were detected in 4-month-old 5XFAD mice. Thus, the present data do not establish an endogenous circulating H_2_S deficit in this model at this early disease stage.

Beyond conventional MWM parameters, a detailed qualitative analysis of swim trajectories revealed genotype- and sex-dependent differences in search strategies. Female Tg mice exposed to sal rely disproportionately on non-cognitive strategies compared with WT females, confirming early AD-related deficits in spatial navigation [56]. Chronic SPA water exposure significantly increases the use of spatially accurate strategies in female Tg mice, nearly tripling the proportion of mice employing effective strategies (from 18% to 57%). In contrast, male mice predominantly employ spatially accurate strategies regardless of genotype or treatment. These findings are noteworthy because they indicate that qualitative analysis of navigation strategies detects subtle functional alterations before deficits become apparent in conventional MWM parameters such as escape latency or probe performance. These observations indicate that H_2_S inhalation selectively enhances the qualitative aspects of spatial navigation particularly in females who exhibit earlier and more severe pathology [27,57,58]. The improvement in spatial search strategies likely reflects early modulation of hippocampal circuitry, synaptic plasticity, or neuroinflammatory pathways, consistent with previous reports that H_2_S can restore sulfhydration of key proteins, reduce neuroinflammation, and enhance synaptic function in AD models [23,48].

We next examined Aβ burden and microglial response following SPA exposure. Notably, SPA treatment significantly reduced the number of 6E10^+^ and 11A1^+^ Aβ plaques in the CTX and DG of Tg mice. While these findings suggest that SPA attenuates Aβ deposition, the present study does not establish the molecular mechanisms responsible for this effect. It is important to note that 6E10 antibody also recognizes full-length APP, β-carboxy-terminal fragments (β-CTFs), and Aβ monomers; therefore, 6E10 IR cannot be interpreted as exclusively reflecting mature amyloid plaques. The parallel reduction observed with 11A1 staining, however, strengthens the conclusion that SPA attenuates Aβ pathology. These observations align with previous findings on the modulatory effects of H_2_S on amyloid pathology in transgenic mouse models of AD [25,42,49,59]. Interestingly, SPA reduced plaque burden across all plaque-size categories without altering their relative distribution, suggesting a global reduction in amyloid deposition rather than a change in the rate of plaque maturation.

The reduction in amyloid pathology was accompanied by attenuation of early cortical microglial response. Although SPA did not significantly attenuate the overall increase in cortical Iba1 IR, it significantly reduced the number of Iba1^+^ cells in Tg mice. In contrast, microglial soma area remained unchanged. The apparent dissociation between Iba1 IR, cell number, and some areas may reflect the fact that these parameters capture different aspects of microglial reactivity. Regional Iba1 IR depends not only on microglial cell number but also on Iba1 expression and the extent and complexity of microglial processes. Thus, the persistence of elevated Iba1 IR despite a reduced number of Iba1^+^ cells may reflect changes in microglial features that were not captured by soma-area measurements. Consistently, plaque-associated microglial clustering was preserved following SPA treatment, suggesting that SPA does not simply suppress the microglial response to amyloid deposition.

Gene expression analysis revealed that SPA exposure selectively modulates pathways associated with microglial responses and cellular homeostasis. *Cst*, *Sod1*, *Tgfb1*, and *Hsp90a* were significantly affected by treatment, while significant genotype × treatment interactions were observed for *Cst*, *Tgfb1*, and *Hsp90a*, indicating a differential response to SPA exposure in Tg and WT mice. *Tgfb1* overexpression has been shown to facilitate Aβ clearance through microglial activation, reducing parenchymal plaque burden and promoting phagocytosis [60]. Similarly, reduced Cst (via genetic variation) has been associated with greater AD risk [61], whereas Cst may exert neuroprotective effects by interacting with Aβ, inhibiting its aggregation, and limits plaque formation [62]. These observations provide a potential biological significance of the SPA-associated modulation of these genes, although their functional contribution to the effects observed in the present study remains to be established.

Mechanistically, these transcriptional changes may reflect the well-established immunomodulatory actions of H_2_S rather than generalized suppression of neuroinflammation. In vitro studies have shown that H_2_S protects microglia from Aβ-induced toxicity [63] and promotes polarization toward a neuroprotective M2 phenotype through CaMKKβ-dependent activation of AMPK, suppressing neuroinflammation [64]. Although microglial polarization was not directly assessed in the present study, the combination of reduced cortical Iba1^+^ cell number, selective modulation of inflammatory genes, and preserved plaque-associated microglial clustering supports the hypothesis that SPA promotes a more homeostatic microglial response during the early stages of AD.

In addition to these significant transcriptional effects, *Cd11b*, *Ccl3*, and *Ccl6* showed a trend toward a genotype-dependent modulation by SPA. Although these changes did not reach statistical significance and should therefore be interpreted cautiously, these genes are involved in microglial responsiveness, chemotactic signaling, and immune-cell recruitment [65]. Their modulation is therefore consistent with, although does not demonstrate, an effect of SPA on early neuroimmune signaling. Overall, these findings suggest that SPA reshapes selected components of the neuroimmune response associated with early Aβ pathology [27]. Whether these changes contribute directly to Aβ uptake or clearance will require specific functional investigation. A limitation of the present study is that, although chronic SPA exposure significantly increased total circulating H_2_S, free serum H_2_S remained unchanged [44]. Moreover, no genotype-dependent differences in circulating H_2_S were detected at this early disease stage. These findings indicate that serum free H_2_S may not accurately reflect biologically relevant H_2_S signaling within the brain and prevent us from establishing whether endogenous cerebral H_2_S metabolism is altered in 4-month-old 5XFAD mice. Direct measurements of brain H_2_S levels, H_2_S-producing enzymes, and H_2_S-dependent protein modifications will therefore be required to address this question. Second, we cannot exclude that the protective effects of SPA-inhalation observed in this study may be related to the presence of minerals and ions naturally present in the sulphureous water, whose beneficial effects have been demonstrated [66,67,68]. Additional studies comparing SPA water with purified H_2_S donors will therefore be necessary to dissect the specific contribution of each component.

Despite these limitations, a major strength of the present study is that it provides the first demonstration that chronic inhalation of naturally H_2_S-rich SPA water ameliorates multiple early hallmarks of AD—including amyloid pathology, cortical microglial reactivity, inflammatory signaling, and subtle behavioral alterations—in a severe AD transgenic model during the prodromal phase of disease. Furthermore, its non-invasive route of administration represents a key translational advantage, as it is expected to promote long-term patient adherence and facilitate its potential clinical implementation.

Finally, emerging evidence broadens the conceptual framework linking H_2_S to AD. H_2_S is increasingly recognized as a “silent neuroprotector,” modulating NMDA receptor-dependent synaptic plasticity and maintaining neurovascular integrity, both disrupted early in AD [69]. Moreover, gut microbiota-derived H_2_S also contributes to systemic sulfur metabolism and neuroinflammatory signaling along the gut–brain axis, with alterations reported in AD and mild cognitive impairment [70]. These observations emphasize that H_2_S dysregulation extends beyond the brain and involves integrated neurovascular, immune, and gut–brain mechanisms. Within this broader framework, the beneficial effects of SPA inhalation observed in the present study reinforce the therapeutic potential of supplementing H_2_S during the earliest stages of AD progression.

## 5. Conclusions

In conclusion, chronic inhalation of H_2_S-rich SPA water during the prodromal phase of AD-like pathology development in 5XFAD mice attenuates early amyloid pathology, reduces cortical microgliosis and selectively modulates immune- and stress-response genes. At the behavioral level, SPA did not alter conventional measures of spatial learning and reference memory, whereas an improvement in spatial search strategy was specifically detected in female Tg mice. Thus, the behavioral response shows a sex-specific component, while the neuropathological effects of SPA were not restricted to females under the present experimental conditions.

Together with the absence of detectable toxicity, these findings support non-invasive SPA-based H_2_S delivery as a potential translational strategy for targeting multiple early pathological events in AD. Its ease of administration, non-invasive nature, and the existing availability of SPA inhalation treatments represent important advantages for clinical translation, particularly in the context of long-term preventive interventions aimed at delaying disease onset or slowing AD progression.

## Figures and Tables

**Table 1:** **List of primers used for RT-qPCR analyses**. For each transcript analyzed in ex vivo experiments, forward (Fw) and reverse (Rv) primer sequences are indicated along with general functional classification and NCBI reference. **Abbreviations**: *Gapdh*: glyceraldehydes-3-phosphate dehydrogenase; *Rps29*: ribosomal protein S29; *Cd11b*: integrin alpha M (*Itgam*); *Cst*: cystatin F (leukocystatin); *Ccl3*: chemokine (C-C motif) ligand 3; *Ccl6*: chemokine (C-C motif) ligand 6; *Hmgb1*: High mobility group box 1; *Il1b*: interleukin 1 beta; *Il4*: interleukin 4; *Il6*: interleukin 6; *Il10*: interleukin 10; *Sod1*: Superoxide dismutase 1; *Tgfb1*: transforming growth factor 1 beta; *Tnfa*: tumor necrosis factor alpha; *Hsp90a*: heat shock protein 90; *Hif1a*: hypoxia inducible factor 1 alpha.

Function	Transcript	NCBI Ref Seq	Primer	Sequence (5′–3′)
Housekeeping genes	*Gapdh*	NM 008084	Fw	CAAGGTCATCCATGACAACTTT
			Rv	GGGCCATCCACAGTCTTCTG
	*Rps29*	NM 009093.3	Fw	TGAAGGCAAGATGGGTCAC
			Rv	GCACATGTTCAGCCCGTATT
Microglial factors	*Cd11b*	NM 001082960.1	Fw	TACCGTCTACTACCCATCTGGC
			Rv	TTGGTGAGCGGGTTCTGG
	*Cst*	NM 009977.3	Fw	CAGGAAGACCATGCATCACCA
			Rv	ATAGAGTCCGCTTCAAGGCAG
Inflammatory mediators	*Ccl3*	NM 011337.2	Fw	CATATGGAGCTGACACCCCG
			Rv	TCTTCCGGCTGTAGGAGAAGC
	*Ccl6*	NM 009139.3	Fw	CTGGCCTCATACAAGAAATGGA
			Rv	TTGGAGGGTTATAGCGACGAT
	*Hmgb1*	NM 010439	Fw	TGGCAAAGGCTGACAAGGCTC
			Rv	GGATGCTCGCCTTTGATTTTGG
	*Il1b*	NM 008361	Fw	GCTTCAGGCAGGCAGTATC
			Rv	AGGATGGGCTCTTCTTCAAAG
	*Il4*	NM 021283.2	Fw	CGGCACAGAGCTATTGATGG
			Rv	CATCCGTGGATATGGCTCCT
	*Il6*	NM 031168	Fw	ACCGCTATGAAGTTCCTCTC
			Rv	CTCTGTGAAGTCTCCTCTCC
	*Il10*	NM 010548.2	Fw	CCAAGCCTTATCGGAAATGA
			Rv	TCTCACCCAGGGAATTCAAA
	*Sod1*	NM 011434.2	Fw	GGGAAGCATGGCGATGAAAG
			Rv	CCCCATACTGATGGACGTGG
	*Tgfb1*	NM 011577.2	Fw	CAAGGGCTACCATGCCAACTT
			Rv	GTTGTGTTGGTTGTAGAGGGC
	*Tnfa*	NM 013693	Fw	GGTTCTGTCCCTTTCACTCAC
			Rv	TGCCTCTTCTGCCAGTTCC
	*Hsp90a*	NM 010480.5	Fw	CGTTACGAGAGCCTGACGGA
			Rv	TCAGGGTTCGGTCCTGTTTG
Apoptotic factors	*Hif1a*	NM 010431.3	Fw	TGAGGCTCACCATCAGTTATTT
			Rv	CAGTCCATCTGTGCCTTCAT

**Table 2:** **Gene expression analysis of microglial markers, inflammatory and apoptotic-related mediators**. RT-qPCR analysis of the CTX of male and female WT and Tg mice sacrificed after an 8-week long sal- or SPA -exposure. The table shows mean and SD of tested genes. Two-way ANOVA was performed with treatment and genotype as factors and sex as covariate; * *p* < 0.05, ** *p* < 0.01, *** *p* < 0.001, # *p* < 0.1. **Abbreviations:** *Gapdh*: glyceraldehyde-3-phosphate dehydrogenase; *Rps29*: ribosomal protein S29; *Cd11b*: integrin alpha M (*Itgam*); *Cst*: cystatin F (leukocystatin); *Ccl3*: chemokine (C-C motif) ligand 3; *Ccl6*: chemokine (C-C motif) ligand 6; *Hmgb1*: High mobility group box 1; *Il1b*: interleukin 1 beta; *Il6*: interleukin 6; *Il10*: interleukin 10; *Sod1*: Superoxide dismutase 1; *Tgfb1*: transforming growth factor 1 beta; *Tnfa*: tumor necrosis factor; *Hsp90a*: heat shock protein 90; Hif1A: hypoxia inducible factor 1 alpha. **Statistical report**: ***Il1b***: Sex: F (1, 22) = 8.310, *p* = 0.010 (*), Genotype: F (1, 22) = 117.245, *p* < 0.001 (***), Treatment: F (1, 22) = 2.361, *p* = 0.142, Treatment × Genotype: F (1, 22) = 0.072, *p* = 0.791; ***Ccl6***: Sex: F (1, 22) = 3.158, *p* = 0.092 (#), Genotype: F (1, 22) = 39.040, *p* < 0.001 (***), Treatment: F (1, 22) = 2.400, *p* = 0.139, Treatment × Genotype: F (1, 22) = 3.412, *p* = 0.081 (#); ***Ccl3***: Sex: F (1, 22) = 0.791, *p* = 0.385, Genotype: F (1, 22) = 16.848, *p* < 0.001 (***), Treatment: F (1, 22) = 2.961, *p* = 0.102, Treatment × Genotype: F (1, 22) = 3.326, *p* = 0.088 (#); ***Hmgb1***: Sex: F (1, 22) = 6.406, *p* = 0.021 (*), Genotype: F (1, 22) = 4.463, *p* = 0.049 (*), Treatment: F (1, 22) = 0.805, *p* = 0.381, Treatment × Genotype: F (1, 22) = 0.003, *p* = 0.960; ***Cd11***: Sex: F (1, 22) = 1.029, *p* = 0.324, Genotype: F (1, 22) = 27.309, *p* < 0.001 (***), Treatment: F (1, 22) = 0.518, *p* = 0.481, Treatment × Genotype: F (1, 22) = 3.960, *p* = 0.062 (#); ***Cst***: Sex: F (1, 22) = 1.642, *p* = 0.216, Genotype: F (1, 22) = 31.573, *p* < 0.001 (***), Treatment: F (1, 22) = 5.671, *p* = 0.028 (*), Treatment × Genotype: F (1, 22) = 7.251, *p* = 0.015 (*); ***Il6***: Sex: F (1, 22) = 0.045, *p* = 0.835, Genotype: F (1, 22) = 2.528, *p* = 0.129, Treatment: F (1, 22) = 0.044, *p* = 0.836, Treatment × Genotype: F (1, 22) = 2.710, *p* = 0.117; ***Tnfa***: Sex: F (1, 22) = 0.007, *p* = 0.934, Genotype: F (1, 22) = 4.191, *p* = 0.056 (#), Treatment: F (1, 22) = 0.061, *p* = 0.808, Treatment × Genotype: F (1, 22) = 1.321, *p* = 0.265; ***Il4***: Sex: F (1, 22) = 0.648, *p* = 0.431, Genotype: F (1, 22) = 1.991, *p* = 0.175, Treatment: F (1, 22) = 0.244, *p* = 0.627, Treatment × Genotype: F (1, 22) = 2.084, *p* = 0.166; ***Il10***: Sex: F (1, 22) = 2.065, *p* = 0.168, Genotype: F (1, 22) = 0.012, *p* = 0.913, Treatment: F (1, 22) = 0.190, *p* = 0.668, Treatment × Genotype: F (1, 22) = 0.275, *p* = 0.607; ***Tgfb1***: Sex: F (1, 22) = 1.415, *p* = 0.250, Genotype: F (1, 22) = 39.650, *p* < 0.001 (***), Treatment: F (1, 22) = 4.510, *p* = 0.048 (*), Treatment × Genotype: F (1, 22) = 8.253, *p* = 0.010 (*); ***Hif1a***: Sex: F (1, 22) = 4.466, *p* = 0.049 (*), Genotype: F (1, 22) = 0.035, *p* = 0.854, Treatment: F (1, 22) = 2.704, *p* = 0.117, Treatment × Genotype: F (1, 22) = 0.142, *p* = 0.710; ***Hsp90a***: Sex: F (1, 22) = 24.778, *p* < 0.001 (***), Genotype: F (1, 22) = 3.308, *p* = 0.086 (#), Treatment: F (1, 22) = 7.017, *p* = 0.016 (*), Treatment × Genotype: F (1, 22) = 5.209, *p* = 0.035 (*); ***Sod1***: Sex: F (1, 22) = 60.134, *p* = 0.718, Genotype: F (1, 22) = 13.360, *p* = 0.002 (**), Treatment: F (1, 22) = 39.147, *p* < 0.001 (***), Treatment × Genotype: F (1, 22) = 2.766, *p* = 0.115.

	WT				Tg							
	sal		SPA		sal		SPA		Statistics			
Target	Mean	SD	Mean	SD	Mean	SD	Mean	SD	Sex	Genotype	Treatment	Genotype * Treatment interaction
*Cd11b*	1.004	0.096	0.901	0.019	1.287	0.250	1.524	0.307	ns	***	ns	#
*Cst*	1.002	0.069	0.778	0.096	4.570	2.498	10.757	5.200	ns	***	*	*
*Ccl3*	1.011	0.171	0.964	0.181	8.618	4.782	20.437	14.673	ns	***	ns	#
*Ccl6*	1.013	0.180	0.861	0.118	6.505	3.899	10.747	4.726	#	***	ns	#
*Hmgb1*	1.001	0.053	0.965	0.051	1.073	0.155	1.041	0.040	*	*	ns	ns
*Il1b*	1.025	0.241	0.713	0.131	3.205	0.673	2.943	0.868	*	***	ns	ns
*Il4*	1.006	0.123	0.900	0.134	1.002	0.200	1.201	0.393	ns	ns	ns	ns
*Il6*	1.173	0.733	0.473	0.191	1.145	0.377	2.064	2.081	ns	ns	ns	ns
*Il10*	1.110	0.543	1.160	0.255	1.246	0.776	1.041	0.318	ns	ns	ns	ns
*Sod1*	1.002	0.070	0.769	0.053	0.846	0.075	0.713	0.063	ns	**	***	ns
*Tgfb1*	1.002	0.067	0.936	0.027	1.362	0.248	1.887	0.422	ns	***	*	*
*Tnfa*	1.035	0.273	0.759	0.428	1.313	0.497	1.746	1.225	**	#	ns	ns
*Hsp90a*	1.008	0.137	0.974	0.188	1.026	0.132	0.835	0.096	***	#	*	*
*Hif1a*	1.003	0.081	0.927	0.080	0.996	0.127	0.953	0.041	*	ns	ns	ns

## Data Availability

Data are contained within the article and Supplementary Files.

## Supplementary Materials

The following supporting information can be downloaded at: https://www.mdpi.com/article/10.3390/brainsci16101069/s1, Figure S1: 6E10+ and 11A1+ plaque size range distribution in the CTX and in the hippocampal DG of sal- and SPA-exposed 5XFAD Tg mice; Figure S2: SPA treatment did not alter microglial Iba+ soma area or plaque-associated microglia clusters in the CTX of Tg mice.

## Abbreviations

3-MST	3-mercaptopyruvate sulfurtransferase
Abs	Antibodies
AD	Alzheimer’s Disease
ANOVA	Analysis of variance
APP	Amyloid precursor protein
Aβ	Amyloid-β
CAT	Cysteine aminotransferase
CBS	Cystathionine β-synthase
Ccl3	Chemokine (C-C motif) ligand 3
Ccl6	Chemokine (C-C motif) ligand 6
Cd11b	Integrin alpha M (Itgam)
CSE	Cystathionine γ-lyase
Cst	cystatin F (leukocystatin)
Ct	Cycle Threshold
CTFs	Carboxy terminal fragments
CTX	Cerebral cortex
DG	Dentate gyrus
FLD	Fluorescence detection
Fw	Forward
Gapdh	Glyceraldehyde-3-phosphate dehydrogenase
H_2_S	Hydrogen Sulfide
Hif1a	Hypoxia inducible factor 1 alpha
Hmgb1	High mobility group box 1
HPLC	High Performance Liquid Chromatography
hr	Hours
Hsp90a	Heat shock protein 90 alpha
IHC	Immunohistochemical
Il1b	Interleukin 1 beta
Il6	Interleukin 6
Il10	Interleukin 10
IR	Immunoreactivity
mAb	Monoclonal antibody
min	Minutes
MWM	Morris Water Maze
NaHS	Sodium Hydrosulfide
PBS	Phosphate-buffered saline
PS1	Presenilin 1
Rps29	Ribosomal protein S29
RT-qPCR	Real-Time quantitative PCR
Rv	Reverse
s	Seconds
sal	Saline
SD	Standard deviation
SDB	Sulfide dibimane
SEM	Standard error of the mean
Sod1	Superoxide dismutase 1
SPA	(Salus Per Aquam) sulphurous water
Tg	Transgenic
Tgfb1	Transforming growth factor 1 beta
Tnfa	Tumor necrosis factor alpha
WT	Wild type
