# Supplementary material for: Chronic Inhalation of Sulphureous Water Mitigates Early Amyloid Pathology and Associated Microglial Changes in 5XFAD Mice
Source: Brain Sci. 2026 Oct 5;16(10):1069. doi: 10.3390/brainsci16101069 (PMC13702382; doi:10.3390/brainsci16101069)
Supplement: Supplementary file 1 [file brainsci-16-01069-s001.zip › brainsci-4565957-supplementary.pdf]

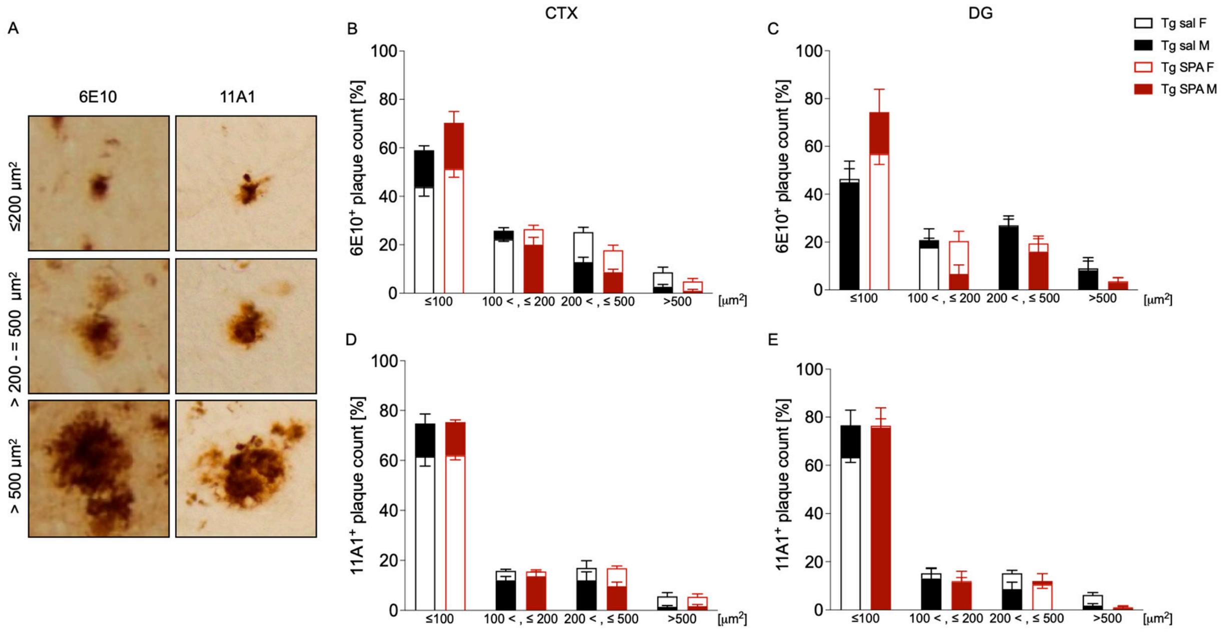

Supplementary Figure S1. 6E10⁺ and 11A1⁺ plaque size range distribution in the CTX and in the hippocampal DG of sal- and SPA-exposed 5XFAD Tg mice. (**A**) Representative 4× images showing analyzed size ranges of 6E10⁺ and 11A1⁺ plaques. (**B**–**E**) % of 6E10⁺ (**B**,**C**) and 11A1⁺ (**D**,**E**) plaques with different size ranges in CTX (**B**,**D**) and DG (**C**,**E**). Data are shown as mean ± SEM. Statistical analysis was performed according to Fisher's exact test comparing size distribution within the same cerebral area (**6E10:** DG: $p = 0.956$, CTX: $p = 0.631$; **11A1:** DG: $p > 0.999$, CTX: $p = 0.984$). **Symbol legend:** Tg sal: $n = 5$ M (filled black bar), 5 F (empty black bar); Tg SPA: $n = 3$ M (filled burgundy bar), 7 F (empty burgundy bar). **Abbreviations:** CTX: cerebral cortex, DG: dentate gyrus, M: male, F: female, sal: saline, SPA: sulphureous water, Tg: transgenic.

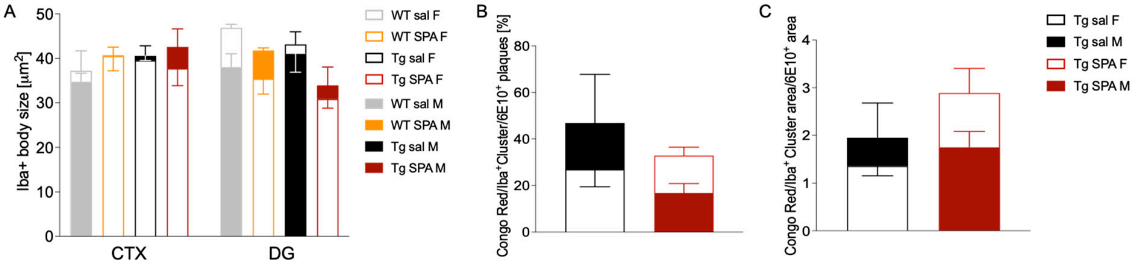

Supplementary Figure S2. SPA treatment did not alter microglial Iba⁺ soma area or plaque-associated microglia clusters in the CTX of Tg mice. (**A**) Iba⁺ microglia body size and (**B**) Congo Red/Iba⁺ cluster number/6E10⁺ plaque number and (**C**) Congo Red/Iba⁺ cluster area/6E10⁺ area in the CTX was quantified in Tg mice treated with sal or SPA. Data are shown as mean ± SEM and compared by two way ANOVA (**A:** Sex: $F(1,27) = 0.075$, $p = 0.787$; Treatment: $F(1, 27) = 1.164$, $p = 0.292$; Genotype: $F(1, 27) = 0.681$, $p = 0.418$; Genotype × Treatment: $F(1, 27) = 1.055$, $p = 0.312$; **B:** Sex: $F(1, 11) = 0.025$, $p = 0.878$; Treatment: $F(1, 11) = 0.954$, $p = 0.354$; **C:** Sex: $F(1, 11) = 0.252$, $p = 0.627$;

Treatment: $F_{(1, 11)} = 1.495$, $p = 0.253$). **Symbol legend:** WT sal: $n = 4$ M (filled grey bar), 4 F (empty grey bar); WT SPA: $n = 4$ M (filled orange bar), 4 F (empty orange bar); Tg sal: $n = 3$ M (filled black bar), 3 F (empty black bar); Tg SPA: $n = 3$ M (filled burgundy bar), 3 F (empty burgundy bar). **Abbreviations:** CTX: cerebral cortex, DG: dentate gyrus, M: male, F: female, sal: saline, SPA: sulphureous water, Tg: transgenic, WT: wild type.
